# Cadmium regulates von Willebrand factor and occludin expression in glomerular endothelial cells of mice in a TNF-α-dependent manner

**DOI:** 10.1080/0886022X.2019.1604383

**Published:** 2019-05-06

**Authors:** Zongguo Sun, Qi Xie, Jie Pan, Na Niu

**Affiliations:** a College of Life Sciences, Shandong Normal University, Jinan, China;; b Medical Research Center, Shandong Provincial Qianfoshan Hospital, Shandong University, Jinan, China;; c Department of Pediatrics, Shandong Provincial Hospital Affiliated to Shandong University, Jinan, China

**Keywords:** Cadmium, von Willebrand factor, occludin, TNF-α, glomerulus

## Abstract

**Background:** Cadmium (Cd) is an environmental pollutant that leads to nephrotoxicity. However, the mechanisms of Cd-induced glomerular injury have not been fully clarified. Von Willebrand factor (vWF) and occludin are important endothelial cell markers in renal vasculature. In this study, the effects of Cd on the vWF and occludin expression in mouse glomeruli was investigated.

**Objectives:** The goal of this study was to analyze the expression of von Willebrand factor and occludin in glomerular endothelial cells of tumor necrosis factor-α^−/−^ (TNF-α^−/−^) mice after treatment with Cd.

**Material and methods:** C57BL6/J wild-type (WT) mice and TNF-α^−/−^ mice (*n* = 6) were treated with Cd, and the kidney tissues were collected. The expression of von Willebrand factor and occludin was detected by using quantitative real-time PCR, immunofluorescence, and immunohistochemistry. *In vitro*, Human umbilical vascular endothelial cells (HUVECs) were used to examine the regulatory role of TNF-α on expression of von Willebrand factor and occludin.

**Results:** We found that Cd significantly increases mRNA and protein expressions of von Willebrand factor and occludin in TNF-α^−/−^ mice, but not in WT mice. *In vitro*, Cd significantly increased mRNA and protein expression of von Willebrand factor and occludin in HUVECs with TNF-α small interfering RNA (siRNA) transfection.

**Conclusions:** These results suggest that TNF-α acts to balance homeostasis of glomerular endothelium after Cd treatments.

## Introduction

Cadmium (Cd) is a heavy metal that is widely used in industry and extensively disseminated in the environment [1]. Occupational exposure, smoking tobacco, and food are main sources of cadmium exposure in humans [2,3]. Once absorbed, Cd mainly accumulates in the liver and kidney after circulation in the blood [4–6]. In the kidney, Cd accumulates in the epithelial cells of the proximal tubule after chronic exposure. When the concentration of Cd reaches 150–200 μg/g tissue, the dysfunction of the proximal tubule is induced, leading to polyuria, low molecular weight proteinuria, and glucosuria [7]. In addition, Cd directly affects glomerular filtration barrier (GFB) [8,9]. The GFB includes glomerular endothelial cells, a unique type of endothelium, the glomerular basement membrane (GBM), and the podocyte [10,11]. Injury to the GFB increases glomerular permeability and proteinuria.

von Willebrand factor (vWF) is a plasma protein almost exclusively expressed by endothelial cells [12]. Before secretion, vWF is stored in Weibel–Palade bodies [13]. vWF is known as a marker of endothelial damage, which is increased in chronic kidney disease (CKD) [14]. The excessive accumulation of ultra-large vWF multimers and platelet aggregation with microthrombus formation induces thrombotic thrombocytopenic purpura, leading to damages of many organs including the kidneys [15].

Occludins and zonula occludens (ZO) proteins are important components of tight junctions (TJs) [16]. Occludin interacts with ZO-1 in increasing the adhesive strength of the tight junction in renal endothelial cells [17]. Occludin is a 65 kDa integral membrane protein, which is indispensable in cortical actin organization and barrier integrity in endothelial cells [18]. Downregulation of occludin leads to elevated endothelial permeability [19].

Cd promotes secretion of proinflammatory cytokines, such as IL-6, IL-8, IL-1β, and tumor necrosis factor-α (TNF-α) [20]. It has been demonstrated that Cd-treated rats had significantly higher tissue TNF-α levels when compared with the control rats [21]. Of note, TNF-α affects the release of vWF from endothelial cells into the circulation [22]. In addition, TNF-α increases epithelial barrier permeability by disrupting tight junctions [5].

To date, the mechanism of Cd injury of renal vasculature has not yet been fully clarified [1,23]. In this study, we investigated the role of Cd on occludin and vWF expression in a mouse kidney. Using TNF-α^−/−^ mice, we determined the effects of Cd on the expression of vWF and occludin is dependent on TNF-α. In HUVECs with TNF-α siRNA transfection, Cd significantly increased mRNA transcription and protein expression of vWF and occludin. This study provided insights for better understanding the mechanisms of Cd injury on glomerular endothelium in the kidneys.

## Materials and methods

### Animals

TNF-α-deficient mice (TNF-α^−/−^) (C57BL6/J background) were purchased from the Jackson Laboratory (005540, Bar Harbor, ME, USA). Wild-type mice (WT) (C57BL6/J background) were purchased from Beijing Vital River Laboratory Animal Technology Co., Ltd. All mice were 6–8-week-old males. TNF-α^−/−^ mice or WT mice were randomly and equally divided into a control group and a trial group, with six mice in each group. Trial group mice were fed a standard chow diet and received treatment with CdCl_2_ (50 mg/L) in distilled water for three consecutive days. The control group was provided water without addition of CdCl_2_ [24]. The mice were maintained under a standard 12-h light/dark cycle, and in conventional housing conditions at 22 ± 1 °C and 50 ± 10% relative humidity. CdCl_2_ was purchased from Sigma Aldrich (St. Louis, MO, USA). All the procedures were approved by the Animal Care Committee of Shandong Provincial Qianfoshan Hospital, Shandong University.

### Enzyme-linked immunosorbent assay (ELISA)

TNF-α^−/−^ mice (*n* = 6) and WT mice (*n* = 6) were injected intraperitoneally with lipopolysaccharide (LPS, 5 mg/kg) and were bled 6 h later. The levels of TNF-α in the serum were determined with an ELISAs Kit (4 A Biotech, Beijing, China) according to the manufacturer’s instructions. LPS was purchased from Sigma Aldrich (St. Louis, MO, USA).

### Quantitative real-time PCR (qRT-PCR)

qRT-PCR was performed as previously described [25,26]. Total RNA was isolated from kidney tissue and HUVECs using the Total RNA Kit I (OMEGA, Norcross, GA, USA) and cDNA synthesis was performed using the RevertAid First-Strand cDNA Synthesis kit (Thermo Fisher, Grand Island, NY, USA). qRT-PCR was carried out using theViiA7 Real-Time PCR System (Applied Biosystems, Waltham, MA, USA). Reaction conditions were as follows: 95 °C for 5 min, 40 cycles of 95 °C for 10 s, and 60 °C for 32 s. All PCRs were repeated in triplicate. Relative expression of genes was calculated using β-actin as an endogenous internal control. The qRT-PCR primer sequences for mouse are summarized in Table 1.

**Table 1.** qRT-PCR Primer sequences for mice.

Gene	Sequence*	Size (bp)	*T*_m_ (ºC)
Occludin	ATTCCATCAGTTTCCTATCT	164	49
	ACCAGGACCTTTCTTGAC		
vWF	GCCCAGGAAGCTATCAGCC	111	57
	ATACACGAAGCCACTCTCGTC		
β-actin	GGATGCAGAAGGAGATTACTGC	94	61
	CCACCGATCCACACAGAGTA		

* All sequences are in the 5’–3’ orientation; bp: base pair; Tm: temperature.

### Immunofluorescence

Immunofluorescence was performed as previously described [27]. Frozen tissue sections (7 μm thickness) were fixed with methyl alcohol. After washing with PBS, sections were incubated with primary antibody vWF (A008229; Dako) and Occludin (71–1500; Invitrogen Camarillo, CA) overnight at 4 °C in a humidified box. After rinsing in PBS, sections were incubated with secondary antibody Alexa Fluor 488 (Thermo Fisher Scientific, USA). Then, sections were incubated with 4′,6-diamidino-2-phenylindole (DAPI) after washing with PBS. Finally, slides were washed with PBS and sealed. Photography was performed with an Olympus FSX100 microscope.

### Immunohistochemistry

The kidney tissue samples from mice were fixed in PBS-buffered 4% paraformaldehyde, dehydrated and embedded in paraffin. The paraffin sections were then dewaxed and dehydrated using alcohol gradient. After thermal remediation, sections were blocked with hydrogen peroxide at room temperature. Then, sections were incubated with primary antibody vWF (Dako, Denmark) and Occludin (Invitrogen Camarillo, CA) overnight at 4 °C. After rinsing with PBS, sections were incubated with secondary antibody Max-Vision TMHRP-Polymer anti-Mouse/Rabbit IHC Kit (Maixin, China).

### Cell culture

Human umbilical vascular endothelial cells (HUVECs) were purchased from American Type Culture Collection (Manassas, VA, USA) and cultured in MV2 media (Promo Cell) with 100 U/mL penicillin and 100 μg/mL streptomycin (Gibco BRL). Cells were maintained in a humidified atmosphere of 5% CO_2_ at 37 °C.

### Transfection

HUVECs were seeded 24 h prior to transfection in 60-mm-diameter dishes with culture media. Transfection was performed with 20 nanomol (nM) TNF-α siRNA and negative control (GenePharma, Shanghai, China) using Lipofectamine 3000 (Invitrogen, Carlsbad, CA, USA) according to the manufacturer's protocol. The next day, media were changed and fresh media containing 4 μM CdCl_2_ were added after 24 h. Cells were collected for RT-PCR after 48 h and for Western blot after 72 h. The qRT-PCR primer sequences for HUVECs are summarized in Table 2.

**Table 2.** qRT-PCR Primer sequences for HUVECs.

Gene	Sequence*	Size (bp)	*T*_m_ (ºC)
TNF-α	CCTCTCTCTAATCAGCCCTCTG	220	63
	GAGGACCTGGGAGTAGATGAG		
Occludin	CCCCATCTGACTATGTGGAAAGA	151	64
	AAAACCGCTTGTCATTCACTGTG		
vWF	CGGCTTGCACCATTCAGCTA	90	64
	TGCAGAAGTGAGTATCACAGCCATC		
GAPDH	TGATGACATCAAGAAGGTGGTGAAG	240	65
	TCCTTGGAGGCCATGTGGGCCAT		

* All sequences are in the 5′–3′ orientation; bp: base pair; *T*_m_: temperature.

### Western blotting

HUVECs were washed with PBS and lysed in RIPA buffer (20 mMTris pH 7.5, 150 mM NaCl, 50 mM NaF, 1% NP40, 0.1% DOC, 0.1% SDS, 1 mM EDTA, 1 mM PMSF, 1 μg/mL leupeptin). Protein concentration was determined using the BCA assay (Bio-Rad, Hercules, CA, USA). Equal amounts of protein were separated by 10% SDS-PAGE gel electrophoresis and transferred to a polyvinylidene fluoride (PVDF) membrane. PVDF membranes were blocked with nonfat dry milk in PBS containing 0.05% Tween-20 and incubated overnight with a primary antibody in PBS-T at 4 °C. Primary antibodies were rabbit anti-human vWF antibody (A008229; 1:800; Dako, Glostrup, Denmark), anti-occludin (13409–1-AP; 1:3000; Proteintech, Wuhan, China), and anti-β-actin (20536–1-AP; 1:8000; Proteintech). Immunoreactivity was visualized with HRP-conjugated secondary antibodies and chemiluminescence (ECL kit, Santa Cruz Biotechnology, Santa Cruz, CA, USA).

### Statistical analysis

The data are expressed as the mean ± standard error. The difference between the two groups was evaluated using the *t-*test (2-tailed). All statistical analyses were performed using SPSS 17.0 statistical software (SPSS Inc., Chicago, IL, USA). A *p* values <0.05 was considered significant.

## Results

### Serum levels of TNF-α were not increased by LPS stimulation in TNF-α^−^^/^^−^ mice

**Figure 1.**
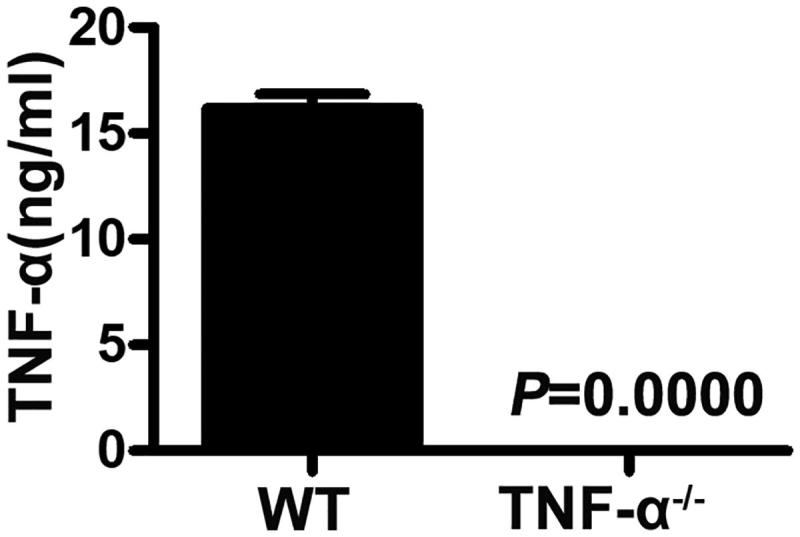
Serum TNF-α levels of mice after LPS stimulation. TNF-α^−/−^ and WT mice (*n* = 6) were injected with LPS. After 6 h, the blood samples were collected from the mice and examined using an ELISA kit for TNF-α (*p* = 0.0000).

To assure that TNF-α was not produced from the TNF-α^−/−^ mice, the TNF-α^−/−^ mice and WT controls were treated with LPS. The blood samples were then collected and TNF-α level was determined using ELISA. Figure 1 shows that the serum level of TNF-α from the WT mice was 16.17 ± 1.7 ng/mL; however, serum levels of TNF-α in the TNF-α^−/−^ mice was almost undetectable (*p* < 0.0001). This suggests that TNF-α expression was successfully eliminated in the TNF-α^−/−^ mice.

### Cd significantly increased gene expression of vWF and occludin in TNF-α^−^^/^^−^ mice

**Figure 2.**
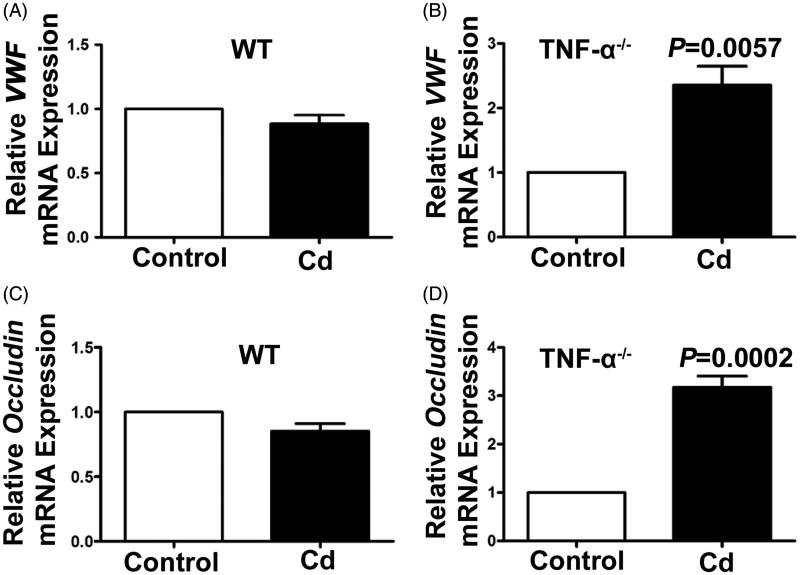
Effects of Cd on vWF and occludin gene expression in TNF-α^−/−^ and WT mice. Relative mRNA expressions of vWF (A) and occluding (C) in the kidneys from WT mice treated with CdCl_2_ for 4 days were determined by using qRT-PCR (*n* = 3). Relative mRNA expression of vWF (B) (*p* = 0.0057) and occludin(D) (*p* = 0.0002) in the kidney from TNF-α^−/−^ mice treated with CdCl_2_ for 4 days were determined by using qRT-PCR (*n* = 3).

To clarify the effect of Cd stimulation on gene expression of vWF and occludin in the kidneys, the TNF-α^−/−^ mice and WT mice were treated with Cd, and the kidney tissues were collected and detected with quantitative real-time PCR. After treatment with Cd, the expression of vWF mRNA was not significantly changed in the WT mice (0.89 ± 0.16-fold, *p* = 0.143, Figure 2(A)) compared to the controls, whereas it was significantly increased in the TNF-α^−/−^ mice (2.36 ± 0.72-fold, *p* = 0.0057, Figure 2(B)). Consistently, the expression of occludin mRNA was not significantly changed in the WT mice (0.85 ± 0.14-fold, *p* = 0.0537, Figure 2(C)); however, it was significantly increased in the TNF-α^−/−^ mice (3.75 ± 0.56-fold, *p* = 0.0002, Figure 2(D)).

### Cd significantly increased vWF and occludin protein expression in glomeruli from TNF-α^−^^/^^−^mice

**Figure 3.**
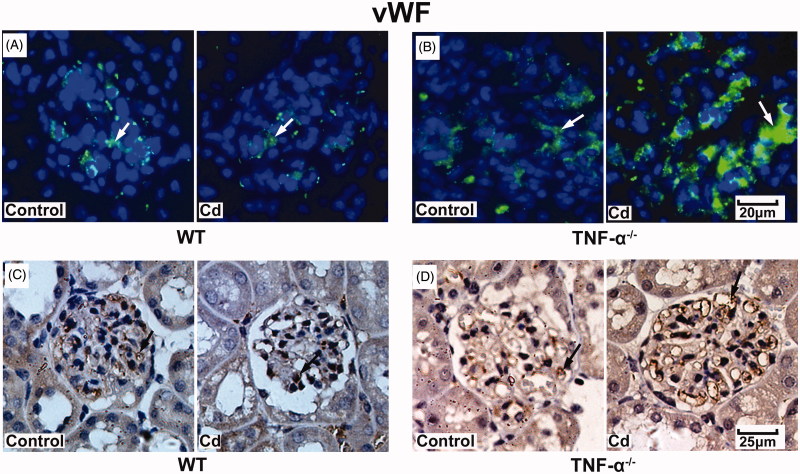
Effects of Cd on vWF protein expression in glomeruli from TNF-α^−/−^ and WT mice. Representative images of vWF immunofluorescent staining in glomeruli of WT mice (A) and TNF-α^−/−^ mice (B). Representative result of vWF expression in WT mice (C) and TNF-α^−/−^ mice (D) using immunohistochemistry.

**Figure 4.**
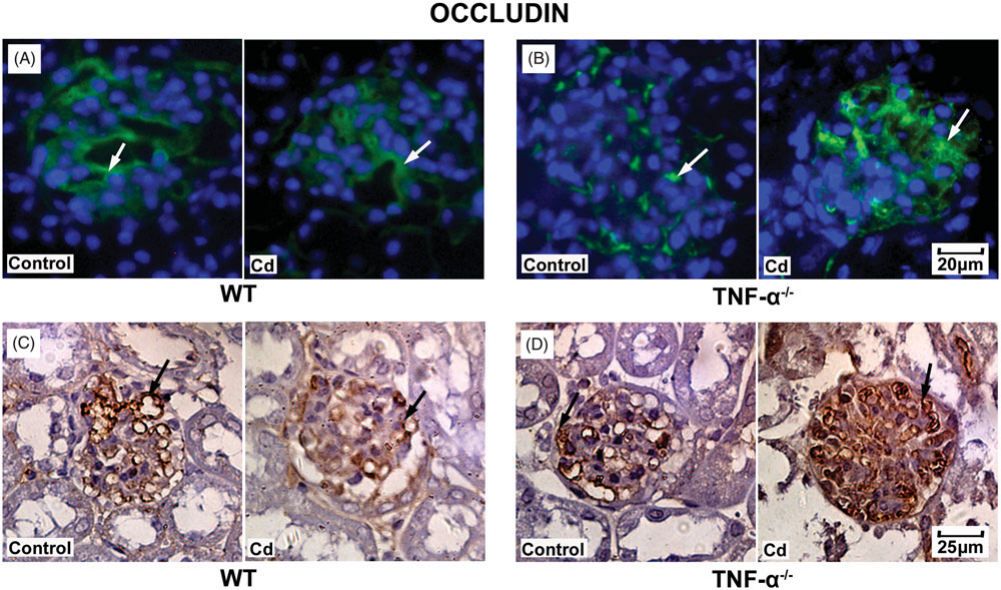
Effects of Cd on occludin protein expression in glomeruli from TNF-α^−/−^ mice and WT mice. Representative result of occludin expression in WT mice (A) and TNF-α^−/−^ mice (B) using immunofluorescence. Representative result of occludin expression in WT mice(C) and TNF-α^−/−^ mice (D) using immunohistochemistry.

To further validate the effects of Cd treatment on protein expression of vWF and occludin in the kidneys, TNF-α^−/−^ mice and WT mice were treated with cadmium and kidney tissues were collected and assessed with immunofluorescence and immunohistochemistry. After treatment with Cd, the expression of vWF was not significantly changed in the glomeruli in the WT mice (Figure 3(A)), whereas it was significantly increased in those of the TNF-α^−/−^ mice (Figure 3(B)). In addition, the expression of occludin was not significantly changed in WT mice (Figure 4(A)), while it was significantly increased in TNF-α^−/−^ mice (Figure 4(B)). Immunohistochemistry was conducted and results were consistent with those from immunofluorescence (Figures 3(D) and 4(D)).

### Cd significantly increased gene expression of vWF and occludin in HUVECs after TNF-α siRNA transfection

**Figure 5.**
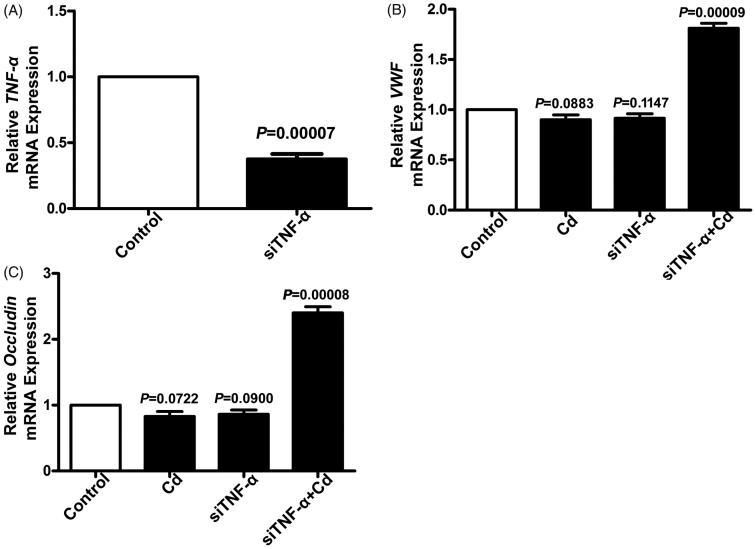
Expression of TNF-α, vWF, and occludin in HUVECs after TNF-α siRNA transfection. (A) Relative mRNA expression of TNF-α in HUVECs was determined using qRT-PCR (*n* = 3, *p* = 0.00007). Relative mRNA expression of vWF (B) and occludin (C) in HUVECs was determined by using qRT-PCR (*n* = 3).

The *in vivo* study showed that the effects of Cd stimulation on gene expression of vWF and occludin might be regulated by TNF-α. *In vitro*, HUVECs were used to validate the regulatory role of TNF-α on expression of vWF and occludin. As shown in Figure 5(A), TNF-α gene expression was significantly downregulated after TNF-α siRNA transfection (*p* = 0.00007, Figure 5(A)). After treatment with Cd, the expression of vWF mRNA was not significantly changed in HUVECs (*p* = 0.0883, Figure 5(B)) compared to the controls, whereas it was significantly increased in TNF-α siRNA-transfected HUVECs (*p* = 0.00009, Figure 5(B)). Consistently, Cd treatment did not change the expression of occludin mRNA in HUVECs (*p* = 0.722, Figure 5(C)), but significantly increased its expression in TNF-α siRNA-transfected HUVECs (*p* = 0.00008, Figure 5(C)).

### Cd significantly increased protein expression of vWF and occludin in HUVECs after TNF-α siRNA transfection

**Figure 6.**
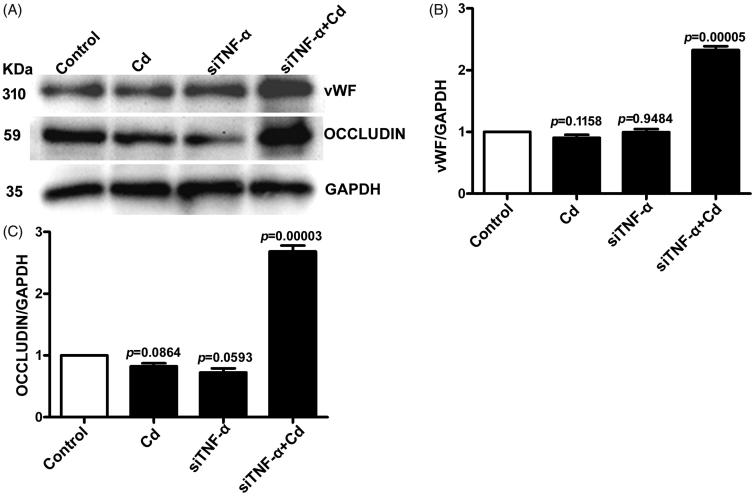
Effects of Cd on vWF and occludin protein expressions in HUVECs after siTNF-α transfection. The nontreatment samples were used as the control and GAPDH was used as loading control. (A) Representative blots of vWF, occludin and GAPDH; (B) Densitometry analysis of vWF/GAPDH; (C) Densitometry analysis of occludin/GAPDH.

To further validate the effect of Cd stimulation on protein expression of vWF and occludin in HUVECs, the treated cells were collected and examined by Western blotting. After treatment of Cd, the protein expression of vWF was unchanged in HUVECs, whereas it was significantly increased in TNF-α siRNA-transfected HUVECs (Figure 6). Consistently, Cd treatment did not change the protein expression of occludin in HUVECs, but significantly increased its expression in TNF-α siRNA-transfected HUVECs (Figure 6).

## Discussion

Cd is a toxic metal that affects mammalian organs and cells [2,28]. Given that little is known about the effect of Cd on the glomerular endothelium, we examined occludin and vWF expression in Cd treated mouse models. In addition, TNF-α^−/−^ mice were used to investigate whether Cd affects the expression of vWF and occludin in the kidney in a TNF-α-dependent manner. The TNF-α^−/−^mice did not produce TNF-α after LPS stimulation, suggesting it as a successful model for TNF-α deletion. Kidney is the primary target of Cd toxicity [29]. Previous studies have demonstrated the protective effect of agents and drugs on Cd-induced nephrotoxicity, such as curcumin [30], ascorbic acid [31], Potentilla anserina polysaccharide [32], and Alpha lipoic acid [33]. Our study provides a new insight into the mechanism of Cd-induced glomerular injury, which may lead to the development of novel treatments.

It has been reported that renal damage is associated with decreased renal expression of occludin [34]. Cd increases endothelial permeability [5] and downregulation of occludin leads to elevated endothelial permeability [35]. The effects of Cd on endothelial cells are dose-dependent [1,36]. In this study we observed that expression of occludin was increased by Cd treatment in TNF-α^−/−^ mice and in TNF-α siRNA-transfected HUVECs, suggesting that TNF-α is involved in inhibition of expression of occludin in the glomerulus. In addition, TNF-α inhibits vWF expression [21,37]. In the absence of TNF-α, Cd significantly increases the expression of vWF. The present study is the first to report that Cd modulates occludin and vWF expression through a TNF-α-dependent manner, indicating the role of TNF-α. in mediating the effects of Cd on glomerular endothelium.

Endothelial cells synthesize and secrete vWF, a key regulator in hemostasis [12]. However, plasma vWF is a risk factor for multiple vascular diseases, including cerebral thrombosis [38], hypertension [39], and coronary disease [40,41]. Cd-induced vWF expression might be involved in these vascular diseases. Inflammatory mediators, such as IL-1, induce the secretion of stored vWF, while other inflammatory mediators, such as TNF-α, act to balance this procoagulant reaction. Both gamma-interferon (IFN-γ) and TNF-α were found to act independently and cooperatively to depress the expression of vWF in endothelial cells [22]. In our study, we found vWF expression was significantly increased by Cd stimulation in TNF-α^−/−^ mice but not in WT mice. In addition, vWF expression was significantly elevated by Cd treatment in TNF-α siRNA-transfected HUVECs. Together, these results suggest that TNF-α regulates vascular homeostasis by decreasing vWF expression during Cd-induced endothelial cell injury.

## Conclusions

In summary, we found that Cd significantly increased expression of vWF and occludin in TNF-α^−/−^ mice and in TNF-α siRNA-transfected HUVECs, suggesting the role of TNF-α in mediating Cd induced toxicity. Our study provides a new insight for understanding the mechanisms of Cd-related effects in glomerular endothelium.

## Abbreviations

CKD: chronic kidney disease
ECs: endothelial cell
IFN-γ: gamma-interferon
GFB: glomerular filtration barrier
GBM: glomerular basement membrane
HUVECs: human umbilical vascular endothelial cells
LPS: lipopolysaccharide
siRNA: Small interfering RNA
TJs: tight junctions
TNF-α: tumor necrosis factor-α
vWF: von Willebrand factor
WT: wild type
ZO: zonula occludens
